# Ionizing radiation-mediated premature senescence and paracrine interactions with cancer cells enhance the expression of syndecan 1 in human breast stromal fibroblasts: the role of TGF-β

**DOI:** 10.18632/aging.100989

**Published:** 2016-07-12

**Authors:** Eleni Liakou, Eleni Mavrogonatou, Harris Pratsinis, Sophia Rizou, Konstantinos Evangelou, Petros N. Panagiotou, Nikos K. Karamanos, Vassilis G. Gorgoulis, Dimitris Kletsas

**Affiliations:** ^1^ Laboratory of Cell Proliferation and Ageing, Institute of Biosciences and Applications, National Centre for Scientific Research “Demokritos”, Athens, Greece; ^2^ Molecular Carcinogenesis Group, Department of Histology and Embryology, Medical School, National and Kapodistrian University of Athens, Athens, Greece; ^3^ Department of Plastic Surgery and Burns Unit, KAT General Hospital of Athens, Greece; ^4^ Biochemistry, Biochemical Analysis & Matrix Pathobiology Research Group, Laboratory of Biochemistry, Department of Chemistry, University of Patras, Patras, Greece; ^5^ Faculty Institute for Cancer Sciences, Manchester Academic Health Sciences Centre, University of Manchester, Manchester, United Kingdom; ^6^ Biomedical Research Foundation, Academy of Athens, Athens, Greece

**Keywords:** senescence, syndecan 1, breast stroma, cancer, TGF-β, ionizing radiation

## Abstract

The cell surface proteoglycan syndecan 1 (SDC1) is overexpressed in the malignant breast stromal fibroblasts, creating a favorable milieu for tumor cell growth. In the present study, we found that ionizing radiation, a well-established treatment in human breast cancer, provokes premature senescence of human breast stromal fibroblasts *in vitro*, as well as in the breast tissue *in vivo*. These senescent cells were found to overexpress SDC1 both *in vitro* and *in vivo*. By using a series of specific inhibitors and siRNA approaches, we showed that this SDC1 overexpression in senescent cells is the result of an autocrine action of Transforming Growth Factor-β (TGF-β) through the Smad pathway and the transcription factor Sp1, while the classical senescence pathways of p53 or p38 MAPK - NF-kB are not involved. In addition, the highly invasive human breast cancer cells MDA-MB-231 (in contrast to the low-invasive MCF-7) can also enhance SDC1 expression, both in early-passage and senescent fibroblasts via a paracrine action of TGF-β. The above suggest that radiation-mediated premature senescence and invasive tumor cells, alone or in combination, enhance SDC1 expression in breast stromal fibroblasts, a poor prognostic factor for cancer growth, and that TGF-β plays a crucial role in this process.

## INTRODUCTION

While the research on cancer development and progression is focusing mainly on neoplastic cells, increasing evidence points to a decisive role of the stroma and its interactions with cancer cells in tumor growth. Indeed, numerous studies indicate that a normal microenvironment can hamper tumor development, while an activated stroma can support tumor progression [1-7]. The stroma is composed of extracellular matrix (ECM) components (such as collagens and proteoglycans) and of many cell types. Among them, fibroblasts play a key role as they are responsible for the deposition and remodeling of ECM constituents, as well as for the release of cytokines and growth factors acting in a paracrine manner on cancer cells [8-11].

Breast represents a classical example where the dynamic interplay between epithelial cells and their microenvironment is crucial for morphogenesis and function, as well as tumor development [12, 13]. Especially during tumor growth, distinct changes have been reported in the breast stroma. Several studies have shown an overexpression of various matrix metallo-proteinases (MMPs), which in many cases have been proposed to be poor prognostic indicators for the disease progression [14-20]. Several proteoglycans are also overexpressed in the breast tumor micro-environment [21], prominent among them being syndecans, a four-member family of transmembrane heparan sulfate proteoglycans (SDC1-4). They are composed of a specific core protein bearing multiple glycosaminoglycan (GAG) chains. Via GAGs they interact with other ECM components, growth factors and cytokines, thus regulating their action. SDC1, 2 and 4 have been reported to be upregulated in breast cancer [21]. Interestingly, detectable SDC1 was also observed in the stroma of malignant tumors and not in the benign breast tissue. It has been proposed that this stromal SDC1 overexpression provides a favorable environment for breast cancer cells' proliferation and migration, as well as angiogenesis, and represents a marker of poor prognosis [21-27].

Normal stromal fibroblasts have a limited lifespan and can undergo replicative senescence after a certain number of cell doublings. This process is the consequence of telomeric DNA shortening, triggering a DNA damage response (DDR), activating the tumor suppressor p53 and subsequently the cyclin-dependent kinase (CDK) inhibitor p21^WAF1^, leading to pRb hypophosphorylation and consequently to a permanent growth arrest [28-30]. Senescent cells also overexpress another CDK inhibitor, i.e., p16^INK4a^, which restrains pRb phosphorylation, as well. In addition, cells exposed to genotoxic insults, such as oxidative stress, UV radiation, or various chemicals, can undergo “stress-induced premature senescence” (SIPS) [28, 31]. SIPS can also be induced by the expression of several proto-oncogenes [32, 33], indicating that this process represents a powerful anticancer mechanism [34]. This is further supported by the presence of senescent cells in a number of premalignant (but not malignant) human and animal tumors [35]. Beyond this beneficial role of senescence as an antitumor barrier, several lines of evidence indicate that senescent cells can support the growth of malignant cancer cells [28, 36]. This is attributed mainly to a specific trait of senescent cells, i.e. the increased secretion of soluble factors such as cytokines, growth factors, and proteases, collectively termed “senescence-associated secretory phenotype” (SASP) [37], disrupting normal architecture and function, thus promoting the growth of malignant and premalignant nearby cells [36, 38, 39]. Several signaling molecules, such as p38 MAPK, NF-kB and p53, have been implicated, in a positive or negative manner, into SASP [37, 40, 41]. However, less attention has been devoted to insoluble factors, i.e. ECM components, although initial observations indicate that such factors are involved in the promotion of tumor growth by senescent stromal cells [36].

Ionizing radiation represents a major means in the treatment of breast cancer. It provokes DNA double strand breaks and inhibits the growth of tumors by inducing cell cycle arrest and/or death of cancer cells. On the other hand, it is a well-known carcinogen in human breast [42]. This is most possibly due to the induction of mutations and/or alterations in the tumor microenvironment [42]. Ionizing radiation inevitably affects stromal cells, too [43] and it can induce a senescence-like state [39, 44]. It has been shown that ionizing radiation-induced senescent human fibroblasts perturb the stromal microenvironment via the increased secretion of MMPs and stimulate the growth of cancer cells *in vitro* and *in vivo* [39, 45]. Here, we studied the effect of ionizing radiation on the induction of premature senescence of human breast stromal fibroblasts *in vitro* and *in vivo*. Furthermore, we investigated the characteristics of the senescent cells, with an emphasis on the expression of SDC1, given that its expression in the stroma can create a favorable environment for tumor cell growth. We also studied the role of the paracrine interactions with human breast cancer cells in SDC1 overexpression in fibroblasts and of the intracellular signaling pathways involved. Particularly, we focused on the autocrine and paracrine action of TGF-β, a factor widely implicated in breast cancer development after irradiation [46, 47].

## RESULTS

### Ionizing radiation-mediated premature senescence in human breast stromal fibroblasts *in vitro* and *in vivo*

**Figure 1:**
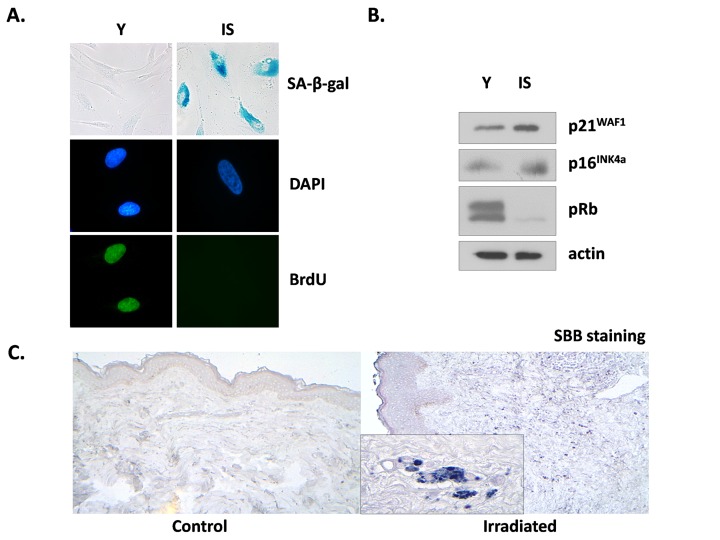
Characterization of irradiation-induced premature senescence in human breast stromal fibroblasts *in vitro* and *in vivo* Early passage (young: Y) and senescent due to irradiation (IS) cells were grown *in vitro* under standard conditions in the presence of 10% (v/v) FBS. In (**A**) cells were stained for SA-β-gal or for BrdU incorporation, while DAPI staining was used for nuclei identification. In (**B**) cell lysates from young and senescent cells were analyzed by western blot for the expression of the indicated proteins. In (**C**) Sudan Black B staining of tissue sections from irradiated vs. non-irradiated (control) human breast tissue from the same individual was performed. One representative experiment out of three similar ones is depicted.

We tested the effects of ionizing radiation in human breast stromal fibroblasts, trying to mimic *in vitro* the conditions prevailing in the stroma during radiotherapy. Thus, we repeatedly exposed confluent non-proliferating fibroblasts to therapeutic doses of radiation (4 Gy) up to a cumulative dose of approx. 50 Gy; alternatively, we exposed the cells in one dose of 50 Gy. The cells were then subcultured and were collected two weeks later, in order to avoid the immediate effects of ionizing radiation. When compared with young (early-passage) proliferating breast fibroblasts, these cells were found to be prematurely senescent as can be seen by the senescent-like morphological alterations, the expression of the senescent marker Senescence-Associated β-galactosidase (SA-β-gal) and the inability for DNA synthesis, shown by the significant decrease of BrdU incorporation (less than 3%, in comparison to more than 70% found in young cells) (Fig. 1A). In addition, in prematurely senescent cells (here called IS cells) overexpression of the cell cycle inhibitors p21^WAF1^ and p16^INK4a^ and absence of the hyper-phosphorylated form of pRb were observed, in accordance with their inability to proliferate (Fig. 1B). Interestingly, the two types of irradiation (repeated low doses or a single high dose) led to identical results (data not shown), as found also in human lung fibroblasts [39]. Accordingly, in all subsequent experiments a single high dose of irradiation was used.

Previous reports indicate that ionizing radiation leads to the prolonged presence of senescence markers, such as DNA damage foci and overexpression of p16^INK4a^ mRNA in several mouse tissues *in vivo*. However, these markers did not coincide with the expression of SA-β-gal positivity, an established marker of senescence [48]. Here we used a novel marker of cellular senescence, i.e. the identification of lipofuscin by Sudan Black B (SBB) staining [49] and as can be seen in Fig. 1C, SBB-positive cells were found in irradiated human breast stroma and not in the non-irradiated breast of the same donor. These data indicate the formation and persistence of senescent cells after irradiation not only *in vitro* but *in vivo*, as well.

### Altered expression of ECM components in senescent human breast stromal fibroblasts

**Figure 2:**
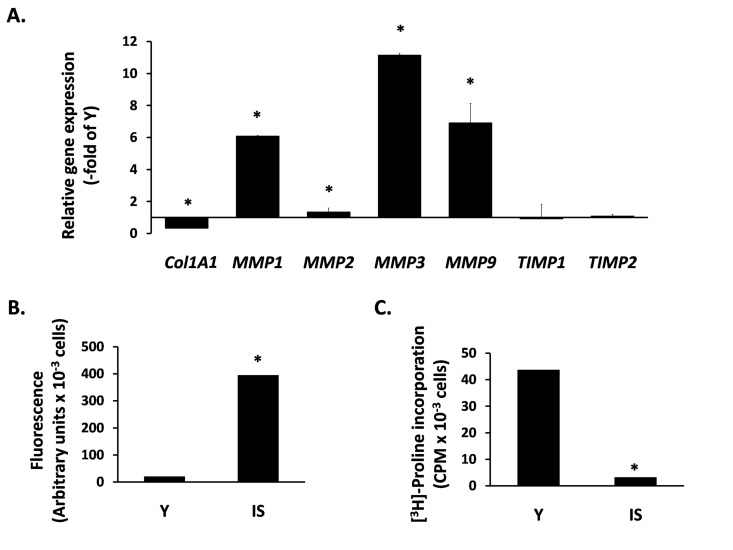
The catabolic phenotype of senescent human breast stromal fibroblasts In (**A**) the expression of genes related to ECM turnover was studied by real-time RT-PCR in senescent due to irradiation (IS) fibroblasts; the expression of each gene is presented relative to its expression in young (Y) cells. In (**B**) MMP activity was measured in the conditioned media of Y vs. IS cells using the TNO211 substrate, while in (**C**) the collagen synthesized by Y and IS fibroblasts was assessed after [^3^H]-proline labeling by the protease-free collagenase method. One representative experiment out of three similar ones is depicted; asterisks indicate statistically significant differences in comparison to Y cells (p < 0.05).

Next, we studied the expression of ECM components in senescent breast fibroblasts. Initially, we studied the transcriptional regulation of MMPs, known to affect breast morphogenesis and to be involved in tumor progression. We found an intense overexpression of several MMPs, i.e. MMP-1, -2, -3 and -9. In addition, we observed a drastic reduction of COL1A1 expression, while the expression of the endogenous tissue inhibitors of MMPs TIMP1 and TIMP2 was unaltered (Fig. 2A). This MMP transcriptional overexpression was further tested at the functional level. By using a FRET-based assay, able to measure the proteolytic activity of all four MMPs mentioned above, it was found that the medium conditioned by senescent cells shows a dramatic upregulation of proteolytic activity, in comparison to the medium conditioned by early-passage cells (Fig. 2B). Finally, we measured the accumulation of collagen secreted by fibroblasts by using the [^3^H] - proline incorporation method that estimates the end-effect of the secretion and/or activation of all the factors mentioned above (collagen, MMPs and TIMPs). As shown in Fig. 2C, collagen accumulation by senescent cells is drastically reduced (compared to young fibroblasts), indicating an intense catabolic phenotype of senescent breast stromal fibroblasts.

**Figure 3:**
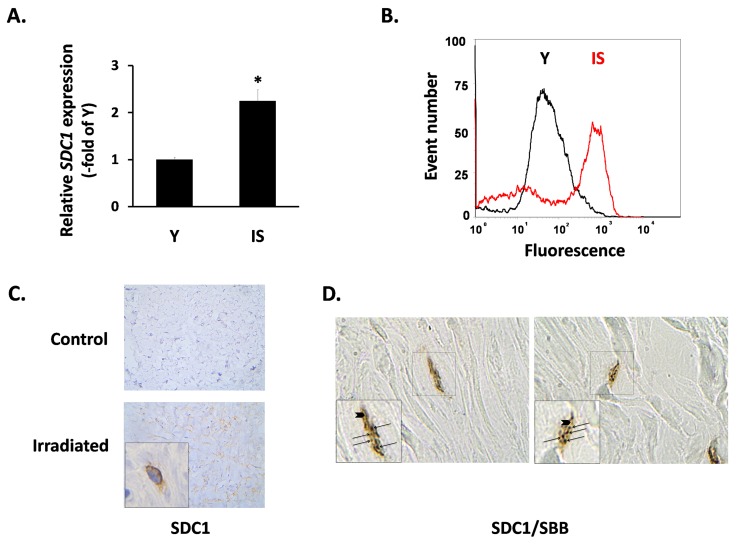
SDC1 expression in senescent human breast stromal fibroblasts *in vitro* and *in vivo* In (A) the expression of the *SDC1* gene in young (Y) and senescent due to irradiation (IS) breast fibroblasts was assessed by real-time RT-PCR; mean values (± standard deviation) of three independent experiments are presented (* indicates p < 0.05 compared to Y cells). In (B) the protein expression of SDC1 on the surface of Y and IS cells was studied after recognition with a specific antibody and flow cytometric analysis (one representative experiment out of three similar ones is presented), while in (C) SDC1 was immunolocalized in tissue sections from irradiated vs. non-irradiated (control) human breast tissue from the same individual. Finally, in (D) cells stained histochemically with Sudan Black B (SBB positive black granules - arrows) and immunohistochemically for Sdc1 (brown color - arrowheads) in irradiated human breast tissue are depicted (Magnification: images x630; inserts x1000).

Subsequently, we focused on the expression of SDC1 in fibroblasts, as its induction in the breast stroma is considered a promoter of tumor growth and a marker of poor clinical outcome [23, 50]. *In vitro*, we found a significant increase of the SDC1 mRNA levels in senescent fibroblasts in comparison to early-passage cells (Fig. 3A). FACS analysis revealed an upregulation of SDC1 at the protein level, as well (Fig. 3B). At the tissue level, we also observed increased SDC1 immunolocalization in irradiated breast stroma and in sites with a significant number of senescent cells, in contrast to the normal breast stroma (Fig. 3C). Even more interestingly, we found increased SDC1 expression in the periphery of SBB-positive (i.e. senescent) cells in samples of irradiated breast stroma (Fig. 3D).

### Invasive breast cancer cells stimulate the upregulation of SDC1 in young and senescent stromal fibroblasts in a paracrine manner: The role of TGF-β

**Figure 4:**
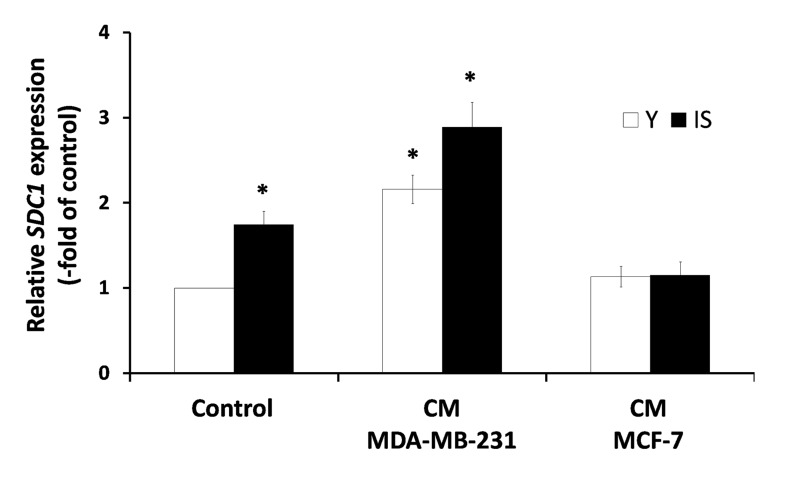
Invasive human breast cancer cells MDA-MB-231 enhance SDC1 expression in stromal fibroblasts in a paracrine mode Early-passage (young: Y) and irradiation-mediated senescent (IS) human breast stromal fibroblasts were incubated with media conditioned by the MDA-MB-231 and MCF-7 human breast cancer cell lines (CM MDA-MB-231 and CM MCF-7, respectively). Twenty four hours later *SDC1* expression was assessed by real-time PCR. One representative experiment out of three similar ones is depicted (* indicates p < 0.05 in comparison to untreated cells).

A previous study, based on a heterologous assay system employing human breast cancer cells and murine embryonic fibroblasts (MEFs), has shown that the highly invasive MDA-MB-231 cells were able to induce SDC1 expression in MEFs, while several low-invasive breast cancer cell lines (e.g. MCF-7) had no effect, whatsoever. Moreover, it has been reported that a direct cell-cell contact was necessary for this effect [25]. Here, we tested the paracrine effect of soluble factors secreted by cancer cells on stromal fibroblasts, in a homologous system, i.e. both cell types (breast cancer cells and stromal fibroblasts) were of human origin. Accordingly, fibroblasts were exposed to media conditioned by the highly invasive MDA-MB-231 or the low-invasive MCF-7 human breast cancer cells. As can be seen in Fig. 4, factors secreted by MDA-MB-231 cells were able to stimulate the expression of SDC1 in young stromal fibroblasts, while MCF-7-derived conditioned medium had no effect. More interestingly, the MDA-MB-231-derived conditioned medium increased even further SDC1 expression also in senescent fibroblasts, while the medium conditioned by MCF-7 was unable to do so (Fig. 4). These data indicate for the first time that aggressive cancer cells and ionizing radiation-induced senescence may synergize to increase SDC1 expression in the breast stroma.

**Figure 5:**
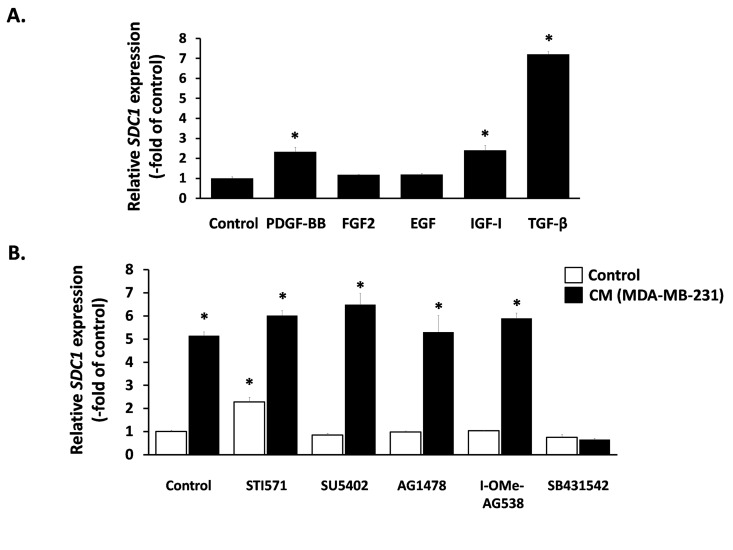
Effect of exogenous and paracrine growth factors on SDC1 expression in human breast stromal fibroblasts (**A**) Early-passage human breast stromal fibroblasts were treated with PDGF (10 ng/ml), FGF2 (10 ng/ml), EGF (100 ng/ml), IGF-I (100 ng/ml) or TGF-β (5 ng/ml) and after 24 h *SDC1* expression was assessed by RT-PCR. (**B**) Cells were pre-incubated for 1 h with growth factor-receptor inhibitors [STI571 for PDGFR (2 μM); SU5402 for FGFR1 (20 μM); AG1478 for EGFR (3 μM); I-OMe-AG538 for IGFIR (12 μM); SB431542 for TGFβR1 (10 μM)]. Then, cells were treated with medium conditioned by MDA-MB-231 (50% v/v), and 24 h later *SDC1* expression was estimated by RT-PCR. One representative experiment out of three similar ones is depicted (* indicates p < 0.05 in comparison to the untreated control).

Next, we aimed at elucidating the nature of this paracrine interaction and we focused on classical growth factors known to induce SDC1 expression in other cell types. We found that TGF-β1 provoked a potent SDC1 stimulation, PDGF-BB and IGF-I had a minor stimulatory effect, while FGF2 and EGF had no effect at all (Fig. 5A). Subsequently, we studied the role of these factors in the paracrine induction of SDC1 by the MDA-MB-231-conditioned medium in breast stromal fibroblasts. To this end, we pre-incubated fibroblasts with specific inhibitors against the surface receptors of the growth factors mentioned above (STI571 for PDGFR, SU5402 for FGFR1, AG1478 for EGFR, I-OMe-AG538 for IGFIR and SB431542 for TGFβR1). As shown in Fig. 5B, all inhibitors acting alone had no inhibitory effect on SDC1 expression in fibroblasts. Furthermore, they were unable to inhibit SDC1 overexpression induced by the MDA-MB-231- conditioned medium, except from SB431542, the specific inhibitor of TGFβR1, which blocked completely this stimulation (Fig. 5B). These data indicate that this paracrine interaction is due to TGF-β secreted by these cancer cells.

**Figure 6:**
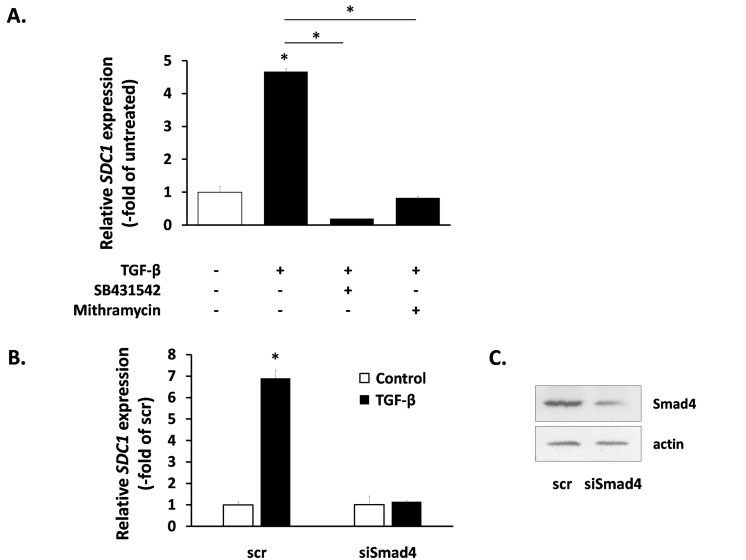
Signaling molecules responsible for SDC1 upregulation by TGF-β in human breast stromal fibroblasts The induction of the *SDC1* gene expression by TGF-β in young human breast stromal fibroblasts was assessed by real-time RT-PCR in the presence or absence of the TGFβR1 inhibitor SB431524 or the Sp1 inhibitor mithramycin (**A**) or after silencing of Smad4 expression by siRNA (**B**); representative experiments out of three similar ones are presented (* indicates p < 0.05 in comparison to the untreated control). In (**C**) the efficiency of Smad4 silencing was assessed by western blotting (one representative experiment is depicted).

We also studied the intracellular signaling pathways used by TGF-β for SDC1 induction. As expected, the SB431542 inhibitor annuls totally the action of TGF-β (Fig. 6A). We also tested the role of the Smad signaling pathway [51] by siRNA-mediated downregulation of Smad4 (the common Smad) (Fig. 6C). As can be seen in Fig. 6B, Smad4 knockdown blocked completely the TGF-β-induced SDC1 up-regulation. Previous reports indicate that the physical interaction and functional cooperation of Smads with the transcription factor Sp1 is important for the expression of many different genes regulated by TGF-β [52], while Sp1 binding sites have been identified in the promoter of the mouse SDC1 gene [53]. According to the above, we found that the specific Sp1 inhibitor mithramycin was able to inhibit entirely the TGF-β-mediated SDC1 induction (Fig. 6A). These data indicate that the Smad pathway cooperates with Sp1 for the SDC1 upregulation by TGF-β.

### The role of TGF-β in the upregulation of SDC1 in senescent stromal fibroblasts

**Figure 7:**
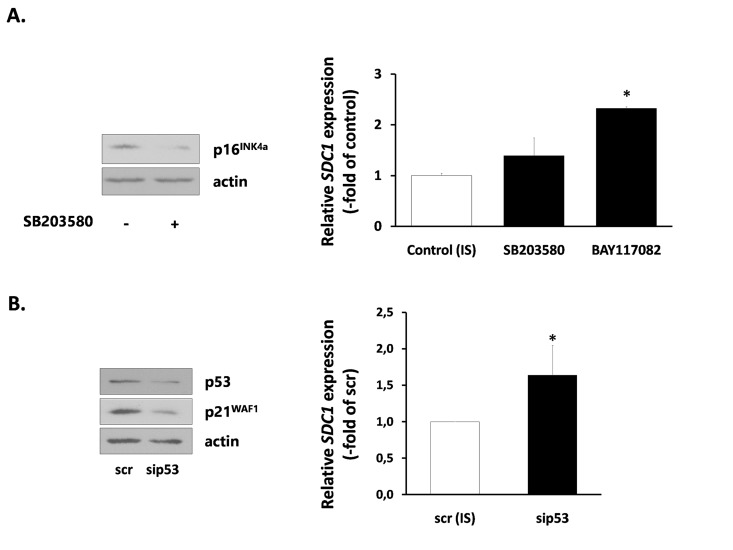
Signaling pathways implicated in SDC1 expression by senescent human breast stromal fibroblasts (**A**) Ionizing radiation-mediated senescent human breast fibroblasts (IS) were treated with the p38 MAPK inhibitor SB203580 or the NF-κB inhibitor BAY117082 (10 μM) and 24 h later *SDC1* expression was assessed by real-time PCR. (**B**) p53 expression was silenced in IS cells by siRNA, as indicated in the Materials and Methods and *SDC1* expression was estimated as in **A**. One representative experiment out of three similar ones is depicted in each case (* indicates p < 0.05 in comparison to the untreated control). In the left panel, the efficiency of SB203580 and p53 siRNA in down-regulating the downstream molecules p16^INK4a^ and p21^WAF1^, respectively, as assessed by western analysis, is depicted.

Finally, we investigated the mechanisms responsible for SDC1 upregulation in senescent breast stromal fibroblasts. We first studied the role of the p38 MAPK - NF-kB axis, known to be responsible for the SASP [40], by using specific inhibitors (SB203580 for p38 MAPK and BAY117082 for NF-kB). SB203580 was able to reduce the expression of the CDK inhibitor p16^INK4a^ in senescent fibroblasts. However, it was unable to reduce SDC1 expression. Even more, BAY117082 was found to increase further SDC1 expression in senescent cells (Fig. 7A). Consequently, we blocked the p53 pathway, also known to control the overexpression of some proteins in senescent cells [41], by using a siRNA approach. Knocking down of p53 resulted, as expected, to a p21^WAF1^ downregulation, but was unable to decrease SDC1 expression (Fig. 7B).

**Figure 8:**
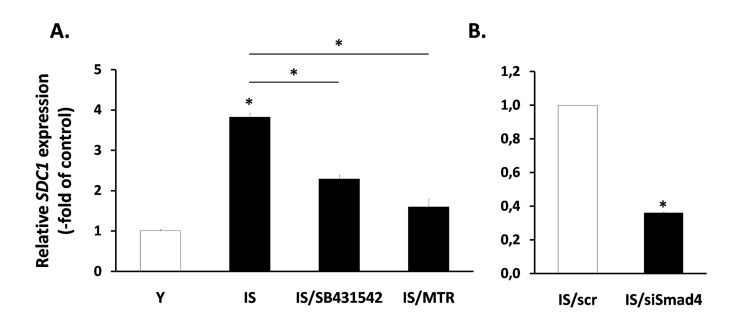
Involvement of the TGF-β signaling pathway in SDC1 overexpression by senescent human breast stromal fibroblasts The levels of the *SDC1* gene expression were assessed by real-time RT-PCR in ionizing radiation-mediated senescent due to irradiation (IS) breast stromal fibroblasts, after treatment in the presence or absence of the TGFβR1 inhibitor SB431524 or the Sp1 inhibitor mithramycin (MTR) (**A**) or after silencing of Smad4 expression by siRNA (**B**). One representative experiment out of three similar ones is depicted (* indicates p <0.05 in comparison to the control).

Next, having in mind the role of TGF-β in the regulation of SDC1 (Fig. 6), we tested its possible involvement in the increased expression of SDC1 in senescent fibroblasts. So, we treated senescent cells with the inhibitors of growth factor receptors mentioned above (see Fig. 5B). Among them, only the TGF-β inhibitor SB431542 was able to significantly reduce SDC1 expression in senescent cells (Fig. 8), in contrast to all other inhibitors tested (data not shown). Interestingly, in early passage cells all inhibitors used (including SB431542) were unable to reduce SDC1 expression (data not shown). Furthermore, we showed that siRNA against Smad4 was able to reduce significantly SDC1 expression in senescent cells (Fig. 8). Finally, and in accordance to the mechanism of TGF-β-mediated SDC1 upregulation described above, the Sp1 inhibitor mithramycin significantly reduced SDC1 expression in senescent fibroblasts. These data suggest that an autocrine loop mediated by TGF-β in senescent cells leads to SDC1 overexpression.

## DISCUSSION

The efficacy of radiotherapy in breast cancer treatment is well established [54]. On the other hand, ionizing radiation can increase the risk of carcinogenesis in human breast and rodent mammary gland due to targeted and non-targeted effects, i.e. DNA mutations and changes provoked to the cancer cells' microenvironment [42]. Indeed, it has been shown that irradiation can lead to changes in the breast stroma milieu, thus creating a “permissive” environment that can contribute to the neoplastic progression [55]. Among the many factors that are involved in these changes TGF-β seems to be of primary importance, known to be involved in the development of mammary microenvironment and tumor progression [46, 56]. Ionizing radiation enhances TGF-β activation in the murine mammary stroma, most possibly after activation of the latent TGF-β complex via the proteolytic system of plasmin or the increased production of reactive oxygen species [57, 58], which precedes the changes observed in the ECM [55]. In addition, radiation does not accelerate tumor development in TGF-β^+/−^ mice, indicating a crucial role of chronic TGF-β presence and activation in radiation-mediated mammary carcino-genesis [47].

Stromal fibroblasts play a key role in tumor development, as they are responsible for the release of cytokines and growth factors acting in a paracrine manner on cancer cells, as well as for the deposition and remodeling of ECM components [8-11]. Normal fibroblasts can undergo senescence after repeated cell doublings or after exposure to various genotoxic stresses and it is well established that cellular senescence represents a potent anticancer mechanism [28, 33, 34]. However, senescent cells once formed are able to promote the growth of cancer cells [59]. Ionizing radiation, a classical genotoxic agent, during radiotherapy can affect not only cancer cells but the adjacent stromal fibroblasts, as well. Here we showed that, similarly to other normal human fibroblast cell strains [36, 38, 39], radiation can drive breast stromal fibroblasts into senescence, as can be seen by the SA-β-gal staining and the overexpression of the cell cycle inhibitors p21^WAF1^ and p16^INK4a^, leading to pRb hypophosphorylation, in agreement with their inability to proliferate. Several lines of evidence indicate that senescent cells express a pro-inflammatory phenotype [28] and consequently senescent cells are eliminated from the tissues *in vivo* by the immune system [60, 61]. On the other hand, it has been shown that ionizing radiation can induce senescence in murine cells *in vivo* several weeks after treatment [48]. These cells are eliminated with different kinetics depending on the tissue of origin, e.g. senescent liver cells are eliminated faster than lung cells. Notably, in this study cell senescence was estimated by the mRNA expression of the senescence marker p16^INK4a^ at the whole tissue level and this did not coincide with the expression of SA-β-gal staining at the single cell level [48]. Here, we verified the presence of senescent cells in irradiated human breast tissue by using an alternative method based on the specific identification of lipofuscin granules with SBB staining [49]. With this staining we were able to identify senescent cells in the irradiated human breast tissue (and not in the non-irradiated tissue of the same individual). This indicates that ionizing radiation provokes premature senescence in human breast stromal fibroblasts not only *in vitro* but also *in vivo* and at least a percentage of these cells remain in the tissue for a considerable amount of time. Further investigation is needed for the elucidation of the kinetics of appearance and elimination of these cells.

Senescent fibroblasts express a specific phenotype characterized by the secretion of several catabolic and pro-inflammatory molecules, such as MMPs, growth factors, inflammatory cytokines and other inflammatory molecules, collectively termed SASP [37]. We have shown that human lung fibroblasts rendered senescent after exposure to ionizing radiation overexpress several MMPs and thus they promote the growth of human lung cancer cells in immunocompromised mice *in vivo* [39]. Here, we also found that ionizing radiation-mediated senescent breast stromal fibroblasts express an intense catabolic phenotype, i.e. increased expression and activity of several MMPs, downregulation of COL1A1, unaltered expression of TIMP1 and TIMP2, ultimately leading to a dramatic reduction of collagen accumulation. This is in accordance with previous observations showing that irradiated breast fibroblasts secrete factors (such as MMPs) affecting mammary ductal morphogenesis and inducing the invasiveness of breast epithelial cancer cells in 3-D culture systems [4, 45].

Although intense attention has been devoted to soluble factors secreted by senescent fibroblasts, less effort has been dedicated to the study of non-soluble factors, such as several components of the ECM. Here we studied especially syndecan family members, known to play a crucial role in breast morphogenesis, tissue repair, inflammation, vascularization and tumor development [21]. We found that irradiation-mediated senescent breast fibroblasts overexpress SDC1, a marker of poor prognosis when expressed in the malignant breast stroma [62] as it alters the assembly of ECM and controls fiber architecture, thus promoting the directional migration of breast cancer cells and facilitating tumor cell spread [25-27]. In this direction, mice with a null mutation in SDC1 are protected from carcinogen-induced tumor development [63]. Accordingly, it seems that senescent cells may add one more alteration (i.e. increased SDC1 expression) in the microenvironment in favor of tumor growth. Finally, we observed that senescent fibroblasts overexpress Sdc4, while Sdc2 expression was unaltered (data not shown here), indicating that there is not any general pattern of syndecans' expression during fibroblast senescence.

It has already been proposed that the upregulation of SDC1 in mouse stromal fibroblasts can be achieved only by highly invasive human breast cancer cells (MDA-MB-231), and that this effect was due to a direct cell-cell contact and not to soluble factors [25]. In contrast to this finding, here we present for the first time evidence that secreted soluble factors (in the form of a conditioned medium) from MDA-MB-231 cells can significantly increase SDC1 expression in human breast stromal fibroblasts. This discrepancy can be possibly explained by the use in our study of a totally homologous cell assay system (human cancer cells acting in a paracrine manner on human stromal fibroblasts), although other reasons cannot be excluded. On the other hand, medium conditioned by the low-invasive MCF-7 breast cancer cells was unable to provoke any SDC1 upregulation. In addition, we showed that the factors secreted from MDA-MB-231 can increase SDC1 expression not only in young fibroblasts, but in senescent fibroblasts, as well. This suggests that a combined action of invasive cancer cells' secreted factors with the effect of irradiation-mediated senescence on the overexpression of SDC1 may promote even further tumor progression.

As the nature of this paracrine interaction leading to SDC1 upregulation in stromal fibroblasts was thus far unknown, we studied the possible role of several growth factors. It has already been reported that PDGF and FGF2 are able to induce SDC1 expression in several mesenchymal cells [64, 65], while EGF and IGF-I were found to be inhibitory [66]. On the other hand, the action of TGF-β was proposed to range from inhibitory to stimulatory in several mesenchymal or epithelial cell strains [65-69]. Here, we studied for the first time the effect of these growth factors on human breast fibroblasts and found that EGF and FGF2 had no effect on SDC1 expression, while PDGF and IGF-I had a moderate stimulatory effect. Finally, TGF-β had the most potent effect as it provoked an intense SDC1 upregulation. Furthermore, we found that TGF-β exerts its action via the classical Smad pathway, as SDC1 upregulation was completely blocked by the down-regulation of the common Smad (i.e. Smad4). In addition, mithramycin, a DNA intercalating agent that interferes with the binding of the transcription factor Sp1, was able to annul the stimulatory effect of TGF-β. This is in accordance with previous data showing that the synergistic cooperation between Smads and Sp1 is crucial for the induction of several TGF-β targets, such as p21^WAF1^, plasmin activator inhibitor-1 [70, 71] and the α2(I)-collagen [72]. In this vein, Sp1 binding sites have been identified in the promoter of the mouse SDC1 gene [53], while, we also found Sp1 binding sites in the promoter of the human SDC1 gene (not shown here). Interestingly, and in agreement with the above, when we used specific inhibitors against the receptors of the above mentioned growth factors we found that the stimulatory effect of the MDA-MB-231-derived conditioned medium on SDC1 upregulation was solely due to TGF-β, since only a specific TGFβR1 inhibitor could block completely this effect, while all the other receptor inhibitors had no effect, whatsoever. In line with the above, a previous report indicated that MDA-MB-231 cells secrete significantly higher TGF-β levels compared to MCF-7, thus possibly explaining the differential effect of the two cell lines on SDC1 expression in breast fibroblasts [73]. This indicates another important involvement of TGF-β on breast cancer development, in addition to the irradiation-mediated TGF-β activation leading to tumor growth.

Among the many phenotypic alterations observed in senescent fibroblasts a major attention has been devoted to SASP. Senescent fibroblasts secrete a large number of catabolic and inflammatory mediators that may affect local tissue homeostasis and even promote the growth of adjacent tumor cells [37]. Although cellular senescence is provoked by a DDR and the activation of the p53 tumor suppression, it has been shown that p53 restrains SASP [37]. On the other hand, SASP is regulated by a DDR-independent activation of the p38 MAPK - NF-kB axis [40, 74]. However, p53 regulates other components overexpressed in senescent cells, like the inflammatory marker ICAM-1 [41]. Having these in mind, we investigated the possibility that these pathways are involved in SDC1 upregulation in senescent breast fibroblasts. Nevertheless, when we blocked p53, p38 MAPK and NF-kB, by siRNA or specific low molecular weight inhibitors, we found that SDC1 expression is not reduced in senescent fibroblasts, indicating that SDC1 overexpression in senescence is not associated with these classical senescence-related pathways. Next, based on our findings that in breast fibroblasts TGF-β is a potent inducer of SDC1, we tested its involvement in SDC1 upregulation in senescent fibroblasts, as well. We found that a specific TGFβR1 inhibitor diminishes significantly SDC1 expression in senescent fibroblasts, indicating the presence of an autocrine loop based on TGF-β. This is in agreement with previous findings indicating an activation of TGF-β in the irradiated breast stroma by plasmin [57]. In the same vein, it has been reported that senescent ulcer skin fibroblasts show elevated production of TGF-β and plasmin [75]. An alternative possibility would be that TGF-β is activated by reactive oxygen species produced by ionizing radiation [58, 76]. In agreement with the presence of a TGF-β-based autocrine loop, SDC1 overexpression in senescent fibroblasts is significantly decreased when the Smad4 pathway is blocked by siRNA, as well as when cells are treated with the Sp1 inhibitor mithramycin, further supporting the role of these two transcription factors in the TGF-β mediated SDC1 overexpression in senescent stromal fibroblasts. Finally, we excluded the possibility that overexpression of SDC1 in senescent cells is due to an increased number of type I and II TGF-β receptors, as young and senescent fibroblasts express similar levels of both receptor types (Suppl. Fig. 1).

**Figure 9:**
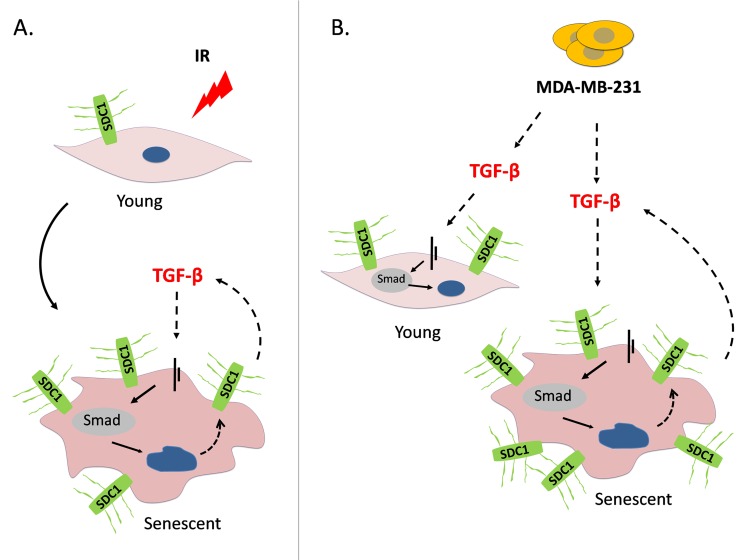
A model depicting SDC1 upregulation in human breast stromal fibroblasts as a consequence of ionizing irradiation-mediated premature senescence and the paracrine action of invasive breast cancer cells (**A**) Ionizing radiation of young (early passage) human breast stromal fibroblasts leads to premature senescence. In senescent cells, an autocrine TGF-β loop is formed leading to SDC1 overexpression via the Smad pathway. (**B**) The highly invasive breast cancer cell line MDA-MB-231 enhances SDC1 expression in young breast fibroblasts in a paracrine manner via the action of TGF-β. Interestingly, it further potentiates SDC1 expression in senescent cells. This indicates a synergistic effect of ionizing radiation with invasive cancer cells leading to the overexpression of SDC1, the latter known to be a poor prognostic factor in breast cancer development.

In conclusion, our data indicate that a genotoxic insult (i.e. ionizing radiation) used in radiotherapy can provoke premature senescence in breast stromal fibroblasts. Under these conditions, an autocrine TGF-β loop is formed leading to SDC1 overexpression, known to facilitate tumor growth and angiogenesis. On the other hand, invasive breast tumor cells stimulate SDC1 in the adjacent young and senescent fibroblasts by secreting TGF-β (Fig. 9). The above suggest the collaboration of a side effect of radiotherapy (i.e. premature senescence) with invasive tumor cells that leads to the upregulation of SDC1, a poor prognostic factor; in addition these data further support the role of TGF-β in breast tumor growth. Interestingly, both cellular senescence and TGF-β have a dual role on tissue homeostasis, as they can both be tumor suppressors in the beginning, while they can play a promoting role after tumor formation [28, 52]. Most probably, the phenomenon described here, i.e. SDC1 overexpression in irradiation-mediated senescent fibroblasts due to TGF-β action, is the combined outcome of the tumor promoting effects of these two factors.

## MATERIALS AND METHODS

### Growth factors and inhibitors

Human recombinant Transforming Growth Factor-beta 1 (TGF-β1) and Platelet-Derived Growth Factor-BB (PDGF-BB) were obtained from Biochrom GmbH (Berlin, Germany). Fibroblast Growth Factor 2 (FGF2) and Insulin-like Growth Factor-I (IGF-I) were purchased from R&D Systems Europe (Abingdon, UK), while Epidermal Growth Factor (EGF) from Sigma (St. Louis, MO, USA). The TGF-β type I receptor (TGFβR1) kinase inhibitor SB431542 was from Sigma, while the PDGF receptor (PDGFR) kinase inhibitor STI571 from Novartis AG (Basel, Switzerland). The FGF receptor 1 (FGFR1) kinase inhibitor SU5402, the IGF-I receptor (IGFIR) kinase inhibitor tyrphostin I-OMe-AG538, and the EGF receptor (EGFR) kinase inhibitor tyrphostin AG1478 were obtained from Calbiochem-Merck KGaA (Darmstadt, Germany). The p38 MAPK inhibitor SB203580, and the NF-κB inhibitor BAY117082 were from Sigma, while the Sp1 inhibitor mithramycin from Cayman Chemical (Ann Arbor, MI, USA).

### Cell culture conditions and induction of ionizing radiation-mediated premature senescence

Human breast cancer epithelial cells MCF-7 and MDA-MB-231 were purchased from the American Type Culture Collection (ATCC, Rockville, USA). Primary cultures of human breast stromal fibroblasts were established after dissection and collagenase (1 mg/ml) digestion of tissues from consenting volunteers undergoing surgery [77]. The study was under the approval of the Bioethics Committee of the NCSR “Demokritos”. Cells were cultured in DMEM supplemented with penicillin-streptomycin, L-glutamine (all from Biochrom) and 10% (v/v) FBS (Gibco BRL, Invitrogen, Paisley, UK). Cells were maintained at 37°C and 5% CO_2_ and subcultured when necessary by using a trypsin/citrate (0.25%:0.30% w/v) solution. In all cases, fibroblasts below population doubling 5 (here named “young” or “early-passage” cells) have been used.

For the induction of premature senescence, human breast stromal fibroblasts grown in 100-mm culture dishes were exposed to γ-irradiation in a ^60^Co gamma source (Gamma Chamber 4000A, Isotope Group, Bhadha Atomic Research Company, Trombay, Bombay, India) at a rate of 3.2 Gy/min and were subcultured 16 h later.

### Collection of conditioned medium

Conditioned medium from subconfluent breast cancer cells was obtained as follows: After 3 washes with PBS, cells grown in 100-mm culture dishes were incubated with phenol red-free and serum-free DMEM for 48 h. Conditioned medium was then collected and centrifuged (400 g / 10 min) to be clarified from cell debris before final storage at −80°C until use.

### Senescence-Associated β-galactosidase (SA-β-gal) staining

Human breast stromal fibroblasts were seeded onto glass coverslips at a density of 1-5 × 10^3^ cells/cm^2^. After 5 days, cells were washed with PBS and fixed with 3% (v/v) formaldehyde for 3 min at room temperature. Cells were then washed again with PBS and incubated with a fresh SA-β-gal staining solution (1 mg/ml X-gal in 40 mM citric acid/sodium phosphate pH 6.0, 5 mM potassium ferrocyanide, 5 mM potassium ferricyanide, 150 mM sodium chloride and 2 mM magnesium chloride) at 37°C for 48 h. SA-β-gal positively-stained cells were observed microscopically.

### Estimation of cell proliferation by BrdU incorporation

For the estimation of cell proliferation, dual labeling with 5-bromo-2′-deoxyuridine (BrdU; Sigma) and 4′,6-diamino-2-phenylindole (DAPI) dihydrochloride (Sigma) was employed. Cells were plated sparsely on glass coverslips and allowed to attach for 48 h before labeling with 50 μM BrdU for another 48 h and fixation with 4% formaldehyde in PBS for 10 min at room temperature. Cell membranes were then permeabilized with 0.2% Triton X-100 in PBS for 10 min and samples were treated with 2N HCl for 30 min at room temperature in order to achieve denaturation of DNA. Following an overnight incubation with anti-BrdU FITC-conjugated antibody from Roche Diagnostics GmbH (Mannheim, Germany) at 4°C, cells were counterstained with 5 μg/ml DAPI in PBS for 20 min at room temperature. DAPI- and BrdU-positive nuclei were visualized under a Zeiss Axioplan 2 fluorescent microscope (Carl Zeiss, Germany).

### Western blot analysis

For total protein extraction cells were washed twice with ice-cold TBS (10 mM Tris-HCl pH 7.4, 150 mM NaCl) and scraped using a hot 2x SDS-PAGE sample buffer [125 mM Tris-HCl pH 6.8, 5% (w/v) SDS, 20% (v/v) glycerol, 125 mM β-mercaptoethanol, 0.02% (w/v) bromophenol blue] supplemented with protease- and phosphatase-inhibitor cocktails (Sigma). Cell lysates were boiled for 3 min, sonicated for 12 sec, clarified by centrifugation, aliquoted and stored at −30°C until use. SDS-PAGE was carried out in Bis-Tris polyacrylamide gels and proteins were transferred to PVDF membranes (Perkin Elmer-Thermo Fisher Scientific, Waltham, MA, USA). Membranes were blocked with 5% (w/v) non-fat milk in 10 mM Tris-HCl pH 7.4, 150 mM NaCl, 0.05% Tween-20 (TBS-T) buffer for 1 h and incubated overnight with the appropriate primary antibodies. The next day membranes were washed 3 times with 5% non-fat milk in TBS-T, incubated with the respective horseradish peroxidase-conjugated antibody for 1.5 h and washed again twice with 5% non-fat milk in TBS-T and once with TBS-T. Immunoreactive bands were visualized on Kodak-X-OMAT AR films by chemiluminescence (ECL kit, GenScript, Piscataway, NJ, USA). At the end of the experiments, membranes were stripped and reprobed with an anti-actin antibody as a loading control. Antibodies against p21^WAF1^, p16^INK4a^ and pRb were from BD Pharmingen (Bedford, MA, USA). Anti-p53, anti-Smad4, anti-TGFβR1 and anti-TGFβR2 were purchased from Santa Cruz Biotechnology (CA, USA), while the panActin antibody was obtained from Neomarkers, Lab Vision Corporation (Fremont, CA, USA).

### Real-time reverse transcriptase polymerase chain reaction (Real-time RT-PCR)

Total RNA was extracted with Trizol reagent (Invitrogen). cDNA for RT-PCR was synthesized from 0.5 μg of total RNA to a total volume of 10 μl, using the PrimeScript RT Reagent Kit (Takara, Tokyo, Japan) according to the manufacturer's instructions. Real time PCR was performed with the obtained cDNA as PCR template at a final dilution of 1:100 using the KAPA SYBR Fast Universal qPCR kit, from KAPA Biosystems (Woburn, MA, USA) with 100 μM of specific reverse and forward primers in a 20 μl reaction. Experiments were conducted in a Mx3000P qPCR Systems Cycler and data analysis was performed with MxPro QPCR Software (Stratagene, La Jolla, USA). Highly purified salt-free primers were generated by VBC Biotech (Wien, Austria) and are presented in Table 1. Relative gene expression was calculated using the 2^−ΔΔCt^ method [78].

**Table 1:** Sequences of the qPCR primers used in this study

Target gene	5′ → 3′ Forward primer	5′ → 3′ Reverse primer
***COL1A1***	CCA GAA GAA CTG GTA CAT CA	CCG CCA TAC TCG AAC TGG AA
***MMP1***	CCT TCT ACC CGG AAG TTG AG	TCC GTG TAG CAC ATT CTG TC
***MMP2***	AAG AAC CAG ATC ACA TAC AGG ATC A	GTA TCC ATC GCC ATG CTC C
***MMP3***	TTT TGG CCA TCT CTC CTT CA	TGT GGA TGC CTC TTG GGT ATC
***MMP9***	CCA CGA CGT CTT CCA GTA CC	TCA ACT CAC TCC GCG AAC TC
***TIMP1***	AAG GCT CTG AAA AGG GCT TC	GAA AGA TGG GAG TCG GAA CA
***TIMP2***	CGA CTG GTC CAG CTC TGA C	ACC CAC AAC CAT GTC TAA AAG G
***SDC1***	AGG ACG AAG GCA GCT ACT CCT	TTT GGT GGG CTT CTG GTA GG
***GAPDH***	GAG TCC ACT GGC GTC TTC	GAG TCC ACT GGC GTC TTC

### Sudan Black B (SBB) and immunohistochemical staining

Staining: SBB specific staining for lipofuscin was performed in formalin-fixed and paraffin-embedded breast tissues, as previously described [49]. Lipofuscin staining was considered positive when perinuclear and cytoplasmic aggregates of blue-black granules were evident inside the cells. Immunohistochemistry was performed in the same samples by using an anti-syndecan 1 antibody (Abcam, Cambridge, MA, USA) at 1:100 dilution, as previously described [49].

Co-staining: For the combined SBB and SDC1 immunohistochemistry we followed the described immunohistochemical method, but after DAB staining, H&E counterstain was omitted and sections were dehydrated until 70% of ethanol. At this point we initiated and completed the above mentioned SBB method except counterstaining with nuclear fast red which was not performed, to avoid any masking effects of the preceded DAB signal.

### Collagen synthesis

A modification of the protease-free collagenase method was employed for collagen synthesis estimation, as described previously [79]. Briefly, breast stromal fibroblasts at approximately 90% confluency were incubated for 24 h in DMEM containing 0.1% (v/v) FBS. Then fresh medium containing 5 μCi/ml L-[^3^H]proline (31 Ci/mmol; Moravek Biochemicals Inc., CA, USA), 50 μg/ml β-aminopropionitrile (Sigma), 50 μg/ml ascorbic acid (Sigma), and 0.1% (v/v) FBS was added for another 48 h. The proteins secreted in the conditioned medium were precipitated with 10% (v/v) trichloroacetic acid (TCA) and re-dissolved in 0.2 N NaOH. One half of each sample was digested with 5 bovine tendon collagen units/ml protease-free collagenase from *Clostridium histoliticum* (EC number 3.4.24.3; Sigma), while the other half was left untreated, to be used as blank. The undigested protein was precipitated with TCA in the presence of tannic acid, and the radioactivity of the supernatant was measured using a β-counter. Collagen synthesis rate for each sample was calculated after subtraction of the blank value and normalization according to the cell number.

### Flow cytometric analysis for the estimation of cell surface SDC1 expression levels

Flow cytometry was employed to measure cell surface SDC1 expression levels as previously described [80]. Cells were harvested with cell dissociation buffer (10mM EDTA in PBS), re-suspended in ice-cold 1% (w/v) BSA in PBS and incubated on ice for 90 min in the presence of an anti-SDC1 antibody obtained from Abcam. Following washing with ice-cold 1% (w/v) BSA in PBS, cells were incubated with a FITC-conjugated IgG secondary antibody (Santa Cruz Biotechnology) for 90 min on ice. Cells were then washed with ice-cold 1% (w/v) BSA in PBS, and finally they were analyzed on a FACSCalibur flow cytometer using the BD CellQuest Pro software (Becton Dickinson, New Jersey, US).

### MMP activity assay

MMP activity of breast stromal fibroblast-conditioned media was determined after addition of the fluorogenic substrate Dabcyl-Gaba-Pro-Gln-Gly-Leu-Glu-(EDANS)-AIa-Lys-NH_2_ (TNO211, Calbiochem) at a final concentration of 7 μM for 24 h at 37°C. TNO211 is substrate for MMP-1, -2, -3, and -9 [81]. Cleavage of the Gly-Leu bond by MMPs, leading to the alleviation of EDANS fluorescence quenching by Dabcyl was monitored at 480 nm after excitation at 340 nm, using an Infinite^®^ M200 microplate reader (Tecan, Männedorf, Switzerland) [81].

### Small interfering RNA (siRNA)-mediated knocking down of human p53 and Smad4

siRNA-mediated knocking down of human p53 and Smad4 was performed as described previously [82, 83]. Human breast stromal fibroblasts were plated and grown in DMEM containing 10% (v/v) FBS for 24 h before siRNA transfection. Cells were transfected with 50 nM of the scramble or the specific siRNA sequence in serum-free OptiMEM I medium using lipofectamine 2000 (Invitrogen) according to the manufacturer's instructions. Five hours later, the transfection medium was aspirated and replaced by DMEM supplemented with 10% (v/v) FBS and cells were incubated for another 72 h. siRNA sequences were purchased from Eurofins MWG Operon (Ebersberg, Germany) and were as follows: 5′-CUACUUCCUGAAAACAACGTT-3′ (for p53 gene silencing), 5′-GGUCUUUGAUUUGCGUCAGTT-3′ (for Smad4 gene silencing) and 5′-UAAUGUAUUGGAACGCAUATT-3′ (predesigned scramble).

### Statistical analysis

Statistical analysis was performed using the Student's t test at a significance level of p < 0.05.

## SUPPLEMENTAL DATA FIGURE


