# Supplementary material for: Ionizing radiation-mediated premature senescence and paracrine interactions with cancer cells enhance the expression of syndecan 1 in human breast stromal fibroblasts: the role of TGF-β
Source: Aging (Albany NY). 2016 Jul 12;8(8):1650–68. doi: 10.18632/aging.100989 (PMC5032688; doi:10.18632/aging.100989)
Supplement: Supplementary file 1 [file aging-08-1650-s001.pdf]

SUPPLEMENTAL DATA

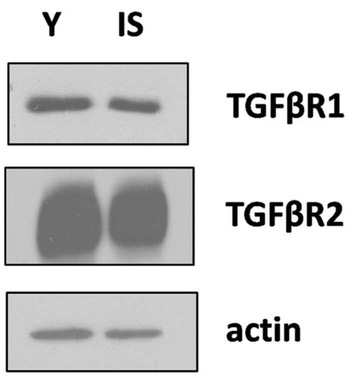

Supplemental Figure 1. Expression levels of TGF-β receptors in human breast stromal fibroblasts. Cell lysates from early passage (young: Y) and senescent due to irradiation (IS) cells were analyzed by western blot for the expression of TGF-β receptor type I (TGFβR1) and type II (TGFβR2). Actin served as reference protein. One representative experiment out of two similar ones is depicted.
